# Posttranslational Modifications and Clearing of α-Synuclein Aggregates in Yeast

**DOI:** 10.3390/biom5020617

**Published:** 2015-04-23

**Authors:** Blagovesta Popova, Alexandra Kleinknecht, Gerhard H. Braus

**Affiliations:** 1 Department of Molecular Microbiology and Genetics, Institute of Microbiology and Genetics, Georg-August-Universität Göttingen, D-37077 Göttingen, Germany; E-Mails: bpopova@gwdg.de (B.P.); ajucker@gwdg.de (A.K.); 2 Center for Nanoscale Microscopy and Molecular Physiology of the Brain (CNMPB), D-37077 Göttingen, Germany

**Keywords:** α-synuclein, Parkinson’s disease, yeast, posttranslational modifications, aggregate clearance, autophagy, proteasome

## Abstract

The budding yeast *Saccharomyces cerevisiae* represents an established model system to study the molecular mechanisms associated to neurodegenerative disorders. A key-feature of Parkinson’s disease is the formation of Lewy bodies, which are cytoplasmic protein inclusions. Misfolded α-synuclein is one of their main constituents. Expression of α-synuclein protein in yeast leads to protein aggregation and cellular toxicity, which is reminiscent to Lewy body containing human cells. The molecular mechanism involved in clearance of α-synuclein aggregates is a central question for elucidating the α-synuclein-related toxicity. Cellular clearance mechanisms include ubiquitin mediated 26S proteasome function as well as lysosome/vacuole associated degradative pathways as autophagy. Various modifications change α-synuclein posttranslationally and alter its inclusion formation, cytotoxicity and the distribution to different clearance pathways. Several of these modification sites are conserved from yeast to human. In this review, we summarize recent findings on the effect of phosphorylation and sumoylation of α-synuclein to the enhanced channeling to either the autophagy or the proteasome degradation pathway in yeast model of Parkinson’s disease.

## 1. Introduction

Parkinson’s disease (PD) is a neurodegenerative disorder of unknown origin that affects approximately 6.3 million people worldwide. The number of new cases increases with age. About 1% of the population older than 60 years and up to 3% older than 85 years is affected [1]. Cases of PD are classified as sporadic or idiopathic where there are no obvious causes (95% occurrence) and familial or genetic-linked (5% occurrence) cases where changes in the genome can be defined. Clinical PD symptoms include slow-downs in motions (bradykinesia) or absence of specific muscle movements (akinesia), resting tremor, and postural instability [2]. These symptoms are attributed to selective loss of dopamine-producing (DA) neurons of the *substantia nigra pars compacta* in the midbrain [3,4]. The reason for the reduction of DA neurons remains unknown. Up to now, neurological damage cannot be reversed and, thus, no cure therapy has been developed for PD.

The pathological hallmark lesions of PD are Lewy bodies (LBs), which represent intraneuronal proteinaceous inclusions. LBs were observed in *post mortem* brain histology [5]. Different α-synuclein point mutations, as well as duplication or triplication of the wild type α-synuclein locus in rare familial forms of PD have been discovered. This is the basis for the current view that α-synuclein plays a key role in the neurodegeneration of PD [6,7,8,9,10,11,12]. The finding of misfolded α-synuclein accumulations as the major constituent of LB in sporadic PD further supported the relevance of α-synuclein for PD [13,14]. LBs containing aggregated α-synuclein were not only found in PD but also in other neurodegenerative diseases as multiple system atrophy, dementia with LB, or Alzheimer’s disease [14,15,16]. This entire group of neurodegenerative disorders has been summarized as α-synucleinopathies.

The small neuronal protein α-synuclein comprises a molecular weight of 14 kDa and is predominantly located at presynaptic terminals and in the nucleus of the central nervous system [17]. The exact function of α-synuclein is not well understood. Several lines of evidence indicate a role in regulation of cell differentiation, synaptic plasticity in presynaptic terminals, dopaminergic neurotransmission, phospholipid metabolism and SNARE complex assembly [18,19,20,21,22,23]. Expression of human α-synuclein in transgenic flies or mice leads not only to inclusion formation, but also to loss of DA neurons resulting in motor deficits [24,25].

The α-synuclein protein is intrinsically unfolded and has the affinity to self-assemble into oligomeric protofibrils. These intermediate forms can further mature into different types of fibrils and insoluble aggregates [26,27]. The familial α-synuclein variants A30P, E46K, and A53T have an increased aggregation propensity *in vitro* and in animal models, but only E46K and A53T enhance the fibrillation *in vivo* and *in vitro* [27,28,29]. A30P mutation exhibits reduced fibrillation *in vitro* in comparison to wild type α-synuclein and other mutants [30]. These and other findings have sparked intense research efforts to understand the mechanism of α-synuclein aggregation and to uncover the correlation between structural features of α-synuclein and its toxicity.

Up to now, it has been controversial which α-synuclein structures might be the pathological species and how the aggregation pathway is initiated. A wide range of factors was identified that promote α-synuclein misfolding or accumulation and contribute to the disease process. This includes mitochondrial dysfunction, oxidative stress, abnormal proteasome function, metals or neurotoxins [31,32,33,34,35,36,37]. In the past few years, a novel concept of prion-like propagation of α-synuclein by cell-to-cell transmission mechanism emerged [38]. Following this hypothesis, *in vivo* studies demonstrated a progressive spreading of α-synuclein between cells with subsequent initiation of “LB-like aggregates” in the acceptor cells [39].

Posttranslational modifications of α-synuclein, such as phosphorylation, ubiquitination, or nitration, were identified at the molecular level to be involved in the α-synuclein aggregation process and result in different impacts on cellular neurotoxicity [40,41,42,43,44]. Phosphorylated α-synuclein was found in brain regions of patients suffering from Alzheimer disease and other synucleinopathies, such as multiple system atrophy, dementia with LB, LB variant of AD, and Hallervorden-Spatz disease [45,46,47,48,49]. The pathology of α-synuclein in other synucleinopathies was reviewed recently [50].

## 2. Yeast as Model for α-Synuclein Aggregation and Toxicity

The budding yeast *Saccharomyces cerevisiae* is a simple unicellular fungus which is lacking any neuron-specific pathways. This eukaryote represents a valuable model system for studying cellular pathways which are required to cope with protein aggregates associated to neurodegenerative diseases. This allows the study of the cellular potential to degrade aggregates. Yeast and humans share many key cellular pathways, such as membrane trafficking, protein aggregation, mitochondrial dysfunction and oxidative stress, transcriptional deregulation, and regulated protein turnover, which are all conserved among eukaryotes [51]. Two-thousand seven-hundred out of 6200 yeast genes (44%) share presumably a common ancestor because at least one conserved domain is similar to human genes with BLAST E values smaller than 10^−10^ [52]. The yeast genome is very well characterized and there are numerous well-established methods available for rapid overexpression or knock-out of almost every gene. Libraries of yeast mutants with gene deletions, conditionally repressible promoters, plasmid overexpression libraries or GFP or TAP-tagged libraries are available, making yeast a suitable platform for systematic genome-scale analysis of cellular processes. More than 80% of approximately 6600 yeast open reading frames are functionally characterized (Saccharomyces Genome Database: http://www.yeastgenome.org). Thus, studies in yeast can be used to identify genes and conserved molecular mechanisms which are relevant and involved in human diseases. Numerous studies have validated yeast as a platform for investigation of α-synuclein cellular toxicity [53]. Expression of α-synuclein in yeast was shown among others to induce oxidative stress [54,55], stimulate the heat shock response [56], affect vesicle trafficking [57,58,59], and induce mitochondrial dysfunction [60,61].

**Figure 1:**
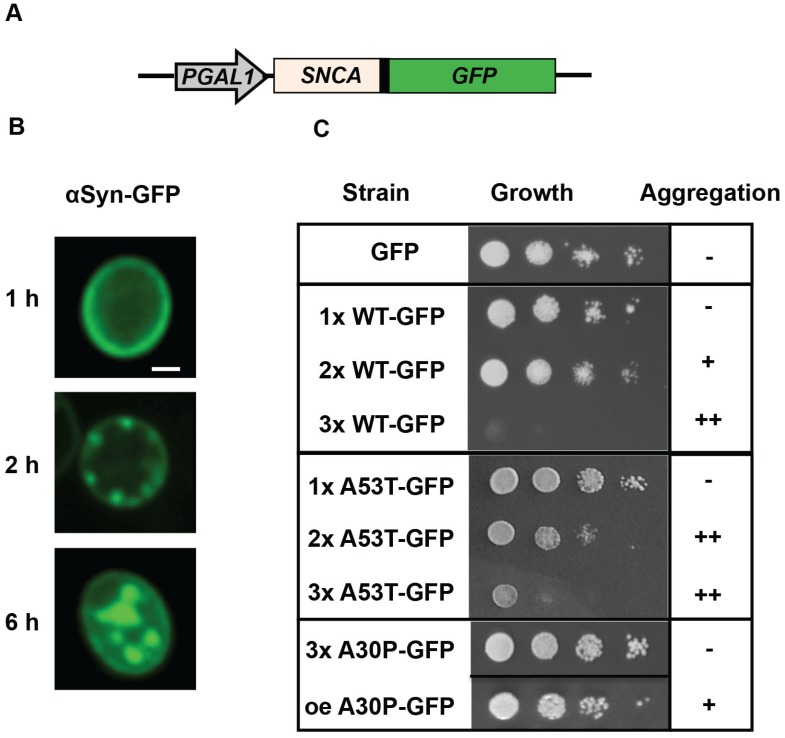
Localization and impact of α-synuclein on yeast cell growth. (**A**) α-synuclein with C-terminally fused GFP is expressed from a regulatable galactose-inducible *GAL1* promoter. GFP does not alter the aggregation behavior or the toxicity of the protein. (**B**) Time-dependent aggregate formation of α-synuclein, expressed from a high-copy plasmid, monitored by fluorescence microscopy. Indicated is the time after induction of expression: (a) early stage (1 h): α-synuclein is localized at the plasma membrane; (b) 2 h—Nucleation at the plasma membrane and formation of small membrane-connected aggregates; (c) late stage (6 h): Formation of large cytoplasmic aggregates by prolonged expression. (**C**) Growth behaviors of yeast cells, carrying increasing number of copies of *GAL1*-driven α-synuclein-GFP fusion alleles with different α-synuclein variants. Spotting analysis indicates decreased growth with increasing copy number of wild-type (WT) and A53T α-synuclein but not A30P. Scale bar = 1 µm; oe, overexpression.

A homologue of the *SNCA* gene encoding human α-synuclein is not present in the yeast genome. However, several relevant aspects of the Parkinson’s disease phenotype are recapitulated when different forms of human α-synuclein or even C-terminally tagged versions are heterologously expressed in yeast cells (Figure 1A; [54,59,62,63,64]). α-synuclein, as well as the mutant variant A53T, which had been discovered in inherited PD patients, are delivered to the yeast plasma membrane through the secretory pathway [56]. This is consistent with the known affinity of α-synuclein for phospholipids. Once the proteins accumulate there, they start to form small seeds that continue to grow in size (Figure 1B). Increase in the expression level of α-synuclein dramatically changes its localization and leads to formation of cytoplasmic inclusions, similar to LBs in neurons of Parkinson patients. This is accompanied with an increase in toxicity, defined as a reduction in cell growth followed by cell death. The increase in α-synuclein mediated toxicity is dose-dependent with a threshold for toxicity (Figure 1C). Three copies of wild-type α-synuclein and two copies of A53T α-synuclein, integrated into a single genomic locus were shown to cause growth inhibition and aggregate formation when the α-synuclein encoding gene was driven by a regulatable house-keeping promoter as the *GAL1* promoter [63]. This promoter is normally required for the catabolism of galactose and is only activated when this sugar is available as nutrient. The response to α-synuclein expression is under these conditions similar to humans, where duplication or triplication of the *SCNA* gene locus driven by its own promoter causes early on-set of PD [9,65].

The A30P α-synuclein variant represents another mutant form identified in familial PD cases. In contrast to wild-type α-synuclein and A53T α-synuclein, A30P mutant revealed only a cytoplasmic localization and does not form aggregates in yeast [56,62]. This is presumably due to the low membrane-binding capacity of A30P α-synuclein that is defective in nucleation of aggregation [64]. A certain degree of A30P aggregate formation had only been described after strong overexpression caused by the high gene dosage of a high copy vector. Aggregate formation of A30P has only a minor effect on yeast cell growth, because aggregation of A30P is only transient [63,66]. This suggests that the yeast cell possesses better clearance mechanisms for A30P in comparison to wild-type or A53T α-synuclein familial variant.

## 3. Posttranslational Modifications of α-Synuclein

α-Synuclein undergoes numerous posttranslational modifications (PTM). These include phosphorylation [46], ubiquitination [45,67], nitration [68], sumoylation [69], glycosylation [70], or acetylation [71]. It is known that PTM of α-synuclein influences its aggregation and toxicity, but the precise contribution of different posttranslational modifications to disease mechanism is still unclear. In this review we focus on recent findings on sumoylation and phosphorylation of α-synuclein and their impact on the protein stability and aggregate degradation in yeast model of Parkinson’s disease.

### 3.1. Sumoylation of α-Synuclein

Sumoylation is a posttranslational modification by which the small ubiquitin-like modifier SUMO is covalently conjugated to target proteins. It is involved in many cellular processes, such as protein stability, nuclear transport, apoptosis, stress response, and transcriptional regulation. Sumoylation occurs by a reversible ATP-dependent process, where SUMO is covalently attached to lysine side chains of target proteins by a thioester bond. Several SUMO isopeptidases can detach the modifier. Therefore, this reversible sumoylation is a highly dynamic process and many proteins go through rapid cycles of sumoylation and SUMO deconjugation [72].

SUMO is expressed by all eukaryotes [73]. Mammals possess at least three SUMO proteins: SUMO1, SUMO2 and SUMO3. The sumoylation pathways of *Drosophila melanogaster* [74], *Caernohabditis elegans* [75] or *Saccharomyces cerevisiae* [76] are essential. There are also multicellular organisms as the mold *Aspergillus nidulans* where a deletion of the SUMO encoding gene is viable but has strong deficiencies in the execution of developmental programs of the organism [77]. *Saccharomyces cerevisiae* possesses only a single SUMO encoding gene (*SMT3*), which corresponds to the SUMO1 encoding gene in mammals, whereas the other human paralogues are missing.

Sumoylation has become increasingly important with regard to neurodegenerative diseases [78,79,80]. Similar to ubiquitin, SUMO co-localizes with the neuronal inclusions associated with several neurodegenerative diseases. This raised the question to identify the SUMO targets within the aggregates. First, it was demonstrated that α-synuclein is monosumoylated by SUMO1 *in vitro* [69]. Another study revealed covalent modification of α-synuclein by SUMO in brain tissues of His_6_-SUMO2 transgenic mice [81]. SUMO conjugation occurred at two major sumoylation sites K96 and K102. It was shown that sumoylation prevented α-synuclein fibril formation *in vitro*. These results suggested that sumoylation negatively regulates α-synuclein aggregate formation by triggering its solubility.

Recently, the yeast model was used to investigate the effect of sumoylation on the cellular toxicity of α-synuclein [82]. It was demonstrated that the cellular mechanism of sumoylation is conserved from yeast to men. Wild-type α-synuclein as well as A30P mutant are sumoylated *in vivo* in yeast at the same sumoylation sites K96 and K102, which had been described as major sumoylation sites in humans. Sumoylation protects yeast cells against α-synuclein-mediated toxicity and inclusion formation. Impairment of sumoylation in yeast resulted in a significant increase in the number of cells with inclusions. Consistently, this correlated with growth inhibition of yeast cells. Amino acid substitution analyses revealed that the protective function of SUMO required direct modification of α-synuclein at the two major sumoylation sites. These findings further confirmed the role of SUMO modification in modulating α-synuclein aggregation and cytotoxicity and support that yeast can be used to study the impact of SUMO on cells which have to cope with high amounts of α-synuclein.

### 3.2. Phosphorylation of α-Synuclein

Phosphorylation of α-synuclein plays an important role in regulation of α-synuclein localization, aggregation and toxicity. Phosphorylation at S129 is the major PTM of α-synuclein as it had been described in human cells. Approximately 90% of the protein found in LB is phosphorylated at this residue, whereas only 4% of the soluble monomeric α-synuclein is phosphorylated at physiological conditions [46,47]. This suggests a close relationship between α-synuclein phosphorylation at S129 and the aggregation propensity of the protein. The A53T and A30P variants are only slightly affected in S129 phosphorylation in comparison to wild-type α-synuclein. In contrast, an E46K variant is significantly increased in S129 phosphorylation in yeast as well as in mammalian cells. This effect is associated to enhanced nuclear and ER accumulation of this variant within the cell [83].

Despite extensive research, there is still no consensus on the effect of phosphorylation on α-synuclein-mediated toxicity and aggregation because the results in different model systems are controversial [84]. Several studies did not observe effects of α-synuclein phosphorylation at S129 on α-synuclein-mediated toxicity and aggregation [85,86]. Studies in *Drosophila melanogaster* [43] and transgenic mouse models [87] revealed pathogenic roles of α-synuclein S129 phosphorylation. Studies in rats [88] and *Caenorhabditis elegans* [89] showed an opposite protective effect of S129 phosphorylation against neuronal dysfunction.

Several kinases participate in α-synuclein S129 phosphorylation in human cells. This includes the Polo-like kinases (PLKs) 1-3, the G protein-coupled receptor kinases (GRKs), the casein kinases (CK) 1 and 2, and the leucine-rich repeat kinase 2 (LRRK2) [49,90,91,92]. PLK2 is the most efficient Polo-like kinase phosphorylating α-synuclein at S129 [93,94,95]. Phosphorylation of α-synuclein by GRK5 plays a crucial role in the pathogenesis of PD [96].

Endogenous kinases phosphorylate α-synuclein also at the conserved S129 site in yeast. The Polo-like kinases and casein kinases possess yeast orthologs. Yeast Polo-like kinase 2 ortholog Cdc5 phosphorylates α-synuclein at S129 and overexpression of Cdc5 rescues α-synuclein toxicity [97,98]. Elevated levels of α-synuclein prevent Cdc5 from maintaining a normal level of GTP-bound Rho1 and thus disrupt the stress-signaling cascade it controls [97]. It was shown that yeast CK-1 and CK-2 casein kinases phosphorylate α-synuclein at S129 and overexpression of yeast Yck3, a vacuolar localized CK-1 kinase, reduces the α-synuclein toxicity [99]. A number of other studies in yeast support the protective role of α-synuclein phosphorylation against toxicity and aggregate formation. Protective role of α-synuclein S129 phosphorylation was described in a strain-specific manner in yeast. Blocking of α-synuclein phosphorylation enhanced the toxicity and trafficking defects in a manner linked to the genetic background [100]. Recent studies in yeast revealed that expression of S129A or S129G variants, which are phosphorylation deficient mutants, promoted the toxicity and α-synuclein inclusion formation [101].

The molecular impact of S129 phosphorylation was further elaborated by heterologous expression of human kinases in yeast and co-expression of α-synuclein and its variants. Expression of PLK2 or GRK5 resulted in a significant increase of α-synuclein phosphorylation at S129 [82]. Increased S129 α-synuclein phosphorylation induced by GRK5 could rescue yeast cells from α-synuclein-mediated cytotoxicity associated with an impairment of sumoylation. Alleviation of the cytotoxicity in SUMO deficient cells correlated with a decreased number of cells with α-synuclein aggregates. Expression of the human kinase GRK5 induced a strong improvement on yeast growth when the sumoylation was impaired either by downregulation of the cellular SUMO pool or by expression of sumoylation deficient α-synuclein mutants. These various yeast studies revealed a complex interplay at the posttranslational level between sumoylation and phosphorylation for protection against α-synuclein toxicity and inclusion formation which is even present in a unicellular eukaryotic cell.

## 4. Degradation Pathways for α-Synuclein

One concept for α-synuclein related toxicity is that increased expression levels due to duplication or triplication of the gene result in a gain of toxic function due to higher amounts of misfolded or aggregated forms of α-synuclein. This is further supported by findings that inefficient protein clearance as a result of impaired degradation pathways is sufficient to trigger neurotoxicity [102,103]. Therefore, the mechanism of α-synuclein aggregate clearance is a central question in understanding the PD pathology. Most cytosolic and misfolded proteins are degraded by the ubiquitin-proteasome system (UPS), which is present in the nucleus and the cytoplasm, or the compartment-linked autophagy and lysosome pathway. The mammalian lysosome corresponds to the vacuole in yeast cells. Impairment of one of these pathways contributes to the accumulation and aggregation of misfolded proteins and cellular toxicity.

Both, UPS and the autophagy/lysosome pathway, contribute to the degradation of α-synuclein [102]. Initial hints that α-synuclein may be degraded by the proteasome came with the identification of ubiquitin and proteasome subunits in LBs of Parkinson patients [104,105]. A number of studies thereafter support that soluble α-synuclein can be a target for the 26S proteasome [106,107]. In contrast, α-synuclein oligomeric forms cannot be subject to proteasomal degradation and rather inhibit the UPS [108,109,110]. Despite the extensive research, the precise role of the UPS in PD pathology and the mechanism of α-synuclein degradation are still unclear.

The involvement of the UPS in α-synuclein toxicity was also investigated in yeast. Yeast cells expressing α-synuclein showed decreased proteasomal function and accumulation of ubiquitin [62]. It was found that the proteasome impairment is due to an altered proteasome composition rather than inhibition of individual peptidases or decreased amount of available proteasome complexes [109]. Inhibition of the UPS activity with the proteasomal inhibitor lactacystin or by using proteasomal mutants revealed increased accumulation of α-synuclein inclusions and enhanced cellular toxicity [56,64,111]. The UPS represents the main degradation pathway under normal *in vivo* conditions.

A different scenario is present for α-synuclein degradation under pathological conditions where the autophagy/lysosome pathway steps in [112]. A number of studies corroborated that in addition to the UPS for monomeric α-synuclein molecules, autophagy represents a major pathway for the degradation of oligomeric species or aggregates of α-synuclein. This also includes the autophagic pathway leading to the lysosome in mammalian cells or to the vacuole in yeast. Chemical inhibition of autophagy promotes α-synuclein accumulation in cultured PD model cells [113]. Autophagy takes care of misfolded proteins when the proteasome becomes impaired. In the presence of an intact UPS, autophagic pathways perform the clearance of large oligomeric forms of α-synuclein, which cannot be degraded by the proteasome [114]. Consistently, the autophagy-activating drug rapamycin stimulates α-synuclein clearance [115] and results in significantly reduced amounts of α-synuclein inclusions [64].

The contributions of the different degradation pathways for clearance of α-synuclein aggregates were further investigated in yeast where only expression of α-synuclein or A53T causes growth inhibition and aggregate formation in a concentration dependent manner. Yeast cells expressing different copies of wild-type α-synuclein or the A30P and A53T variants controlled by the inducible *GAL1* promoter were compared. The production of α-synuclein can be induced in a first phase and then stopped by repression of the *GAL1* promoter. Yeast cells are able to clear aggregates and to regain growth. The contribution of the UPS or the autophagy/lysosome pathways responsible for α-synuclein aggregate clearance can be studied by genetic mutants or by pharmacological treatment with the proteasome inhibitor MG132 or vacuolar proteases inhibitor PMSF. Inhibition of the proteasome does not inhibit aggregate clearance in yeast cells. All results support that in yeast the major contribution to aggregate clearance depends on the vacuolar pathway. The autophagy-monitoring experiments revealed that α-synuclein exerts an inhibitory effect on autophagy induction, yet the molecular mechanism of inhibition has to be further elucidated [63]. The similar finding that α-synuclein inhibits autophagic pathways in neuronal cells [116] suggests that autophagic inhibition by α-synuclein is conserved from yeast to higher organisms.

## 5. Role of Posttranslational Modifications for α-Synuclein Aggregate Clearance

Posttranslational modifications modulate the degradation of aggregation prone proteins by various proteolytic pathways. PTMs presumably act as molecular switches that determine the faith of the protein and its preference for a certain proteolytic pathway. Monoubiquitination is one trigger of α-synuclein which controls the partitioning of the protein between the proteasomal and autophagy systems. Monoubiquitinated α-synuclein is degraded preferentially by the proteasome, whereas deubiquitinated α-synuclein is targeted to the autophagy pathway [117]. Another trigger is phosphorylation. Overexpression of the PLK2 kinase increases α-synuclein phosphorylation and mediates the selective clearance of α-synuclein through autophagic degradation and accordingly causes reduced α-synuclein toxicity [91].

**Figure 2:**
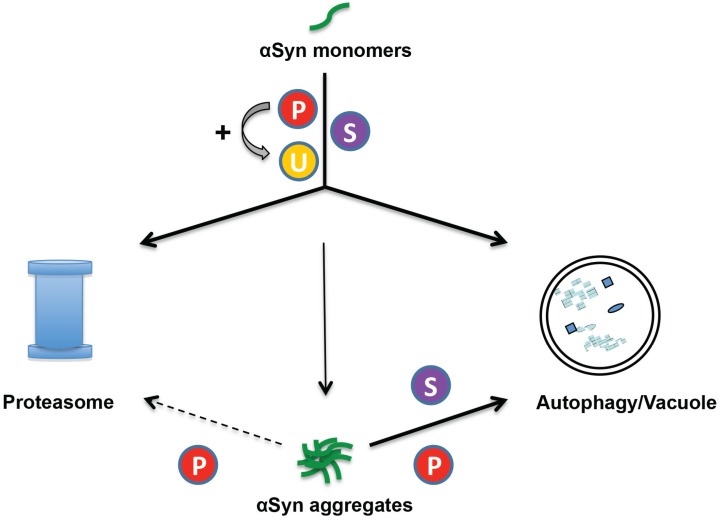
Model of clearance pathways for monomers or aggregates of α-synuclein with different posttranslational modifications in yeast. The proteasome and autophagy/vacuole are the two major pathways for degradation of intracellular proteins. Degradation of soluble α-synuclein monomers occurs through both pathways. Inhibition of α-synuclein sumoylation has a strong effect on monomer protein stability, significantly increasing the half-life of the protein and inhibiting the degradation through both pathways. Phosphorylation at S129 promotes degradation of soluble α-synuclein through proteasome and autophagy pathways. Phosphorylation at S129 additionally promotes α-synuclein ubiquitination and decreased the stability of the protein. When the synthesis of α-synuclein is switched-off, wild-type yeast cells clear α-synuclein aggregates within hours and regain normal growth rates. The main pathway for α-synuclein aggregate clearance is autophagy. Phosphorylated or sumoylated α-synuclein is primarily targeted to the autophagy pathway. Inhibition of sumoylation results in inefficient autophagy-mediated aggregate clearance. Increase of S129 phosphorylation level by GRK5 or PLK2 expression rescues the autophagic aggregate clearance and additionally promotes the proteasomal degradation. S = sumoylation; P = phosphorylation; U = ubiquitination.

Studies in yeast as PD model suggest that there is a complex interplay between different PTMs as triggers for different degradation pathways (Figure 2). The distribution of α-synuclein to different cellular clearing pathways does not only depend on monomeric *versus* multimeric forms but depends also on PTMs. This includes a complex cross-talk between sumoylation of α-synuclein and S129 phosphorylation which affects clearance of α-synuclein aggregates [82]. Sumoylation of α-synuclein promotes aggregate clearance by autophagy in yeast. This clearance is impaired when sumoylation is inhibited either by reducing the cellular SUMO pool or by amino acid substitutions of the SUMO target sites of α-synuclein. Inhibition of sumoylation resulted not only in inefficient autophagy-mediated aggregate clearance but also in redirection of the protein to the proteasome. Sumoylated α-synuclein is, therefore, primarily targeted to the autophagy pathway whereas non-sumoylated α-synuclein is primarily channeled to the proteasome. Expression of human kinases as GRK5 can also promote the clearance of non-sumoylated α-synuclein through the autophagosome as through the proteasome in yeast. The UPS response is increased because phosphorylation at S129 promotes increased α-synuclein ubiquitination resulting in a destabilization. Consistently, blocking of α-synuclein S129 phosphorylation impairs the turnover of α-synuclein in yeast. The expression of a S129A mutant variant promotes α-synuclein toxicity and inclusion formation and slows down the cellular clearance mechanisms [101]. Future studies will show which other molecular cross-talks beside the sumoylation/phosphorylation link might also be used by eukaryotic cells to trigger and distribute α-synuclein to autophagic or proteasomal pathways for degradation.

## 6. Conclusions

Protein quality control mechanisms play an essential role for the accumulation of misfolded and oligomeric protein species in neurodegenerative diseases. The equilibrium between synthesis and degradation of α-synuclein determines the protein level as important determinant of PD progression. Posttranslational modifications of α-synuclein, such as phosphorylation, sumoylation, and ubiquitination, are prominent in PD and are primarily involved in α-synuclein aggregation and clearance. A deeper understanding of the interplay between α-synuclein modifications and degradation pathways is needed. Insights in the involved molecular mechanisms will open new therapeutic strategies for Parkinson patients because they enable a more specific targeting of α-synuclein to cellular clearing pathways.
